# A Breast Cancer Polygenic Risk Score Validation in 15,490 Brazilians Using Exome Sequencing

**DOI:** 10.3390/diagnostics15091098

**Published:** 2025-04-25

**Authors:** Flávia Eichemberger Rius, Rodrigo Santa Cruz Guindalini, Danilo Viana, Júlia Salomão, Laila Gallo, Renata Freitas, Cláudia Bertolacini, Lucas Taniguti, Danilo Imparato, Flávia Antunes, Gabriel Sousa, Renan Achjian, Eric Fukuyama, Cleandra Gregório, Iuri Ventura, Juliana Gomes, Nathália Taniguti, Simone Maistro, José Eduardo Krieger, Yonglan Zheng, Dezheng Huo, Olufunmilayo I. Olopade, Maria Aparecida Azevedo Koike Folgueira, David Schlesinger

**Affiliations:** 1 Mendelics, Sao Paulo 02511-000, SP, Brazil; 2 Comprehensive Center for Precision Oncology (C2PO), Centro de Investigação Translacional em Oncologia (CTO), Departamento de Radiologia e Oncologia, Instituto do Cancer do Estado de Sao Paulo (ICESP), Hospital das Clinicas HCFMUSP, Faculdade de Medicina, Universidade de Sao Paulo, Sao Paulo 01246-000, SP, Brazil; 3 Instituto D’Or de Pesquisa e Ensino (IDOR), Sao Paulo 01401-002, SP, Brazil; 4 Instituto do Coração, Hospital das Clinicas HCFMUSP, Faculdade de Medicina, Universidade de Sao Paulo (FMUSP), Sao Paulo 05403-900, SP, Brazil; 5 Medicine and Human Genetics, Center for Clinical Cancer Genetics and Global Health, University of Chicago Medical Center, Chicago, IL 60637, USA; 6 Department of Public Health Sciences, University of Chicago, Chicago, IL 60637, USA

**Keywords:** polygenic risk score (PRS), breast cancer, admixed population, cancer genetics, next-generation sequencing (NGS), precision medicine

## Abstract

**Background/Objectives**: Brazil has a highly admixed population. Polygenic risk scores (PRSs) have mostly been developed from European population studies, and their application to other populations is challenging. To assess the use of PRS for breast cancer (BC) risk in Brazil, we evaluated four PRSs in the Brazilian population. **Methods:** We analyzed a Brazilian cohort composed of 6206 women with a history of breast cancer and 8878 unphenotyped adults as controls. Genomic variants were imputed from exomes, and scores were calculated for all samples. **Results:** After individuals with known pathogenic or likely pathogenic variants in *BRCA1*, *BRCA2*, *PALB2*, *PTEN*, or *TP53* genes, and first-degree relatives of the probands were excluded, 5598 cases and 8767 controls remained. Four PRS models were compared, and PRS_3820_ achieved the best performance, with an odds ratio (OR) of 1.43 per standard deviation increase (*p* value < 0.001) and an OR of 1.88 (*p* value < 0.001) for the top decile. PRS_3820_ also performed well for different ancestry groups: East Asian majority (OR 1.59, *p* value 0.004), Non-European majority (OR 1.45, *p* value < 0.001), and European majority (OR 1.43, *p* value < 0.001). **Conclusions:** Among the different PRSs, PRS_313_ and PRS_3820_ could be validated in our Brazilian cohort, with the latter exhibiting the best performance. While further clinical studies are necessary to guide clinical practice, this work represents an important step toward improving BC precision medicine in Brazil.

## 1. Introduction

Breast cancer (BC) is a critical global health concern and represents the most commonly diagnosed cancer among women worldwide [1]. In Brazil, more than 70,000 women are diagnosed with BC annually, accounting for roughly 30% of all cancers in the female population [2].

Lifestyle, environmental, and genetic factors collectively contribute to BC risk, characterizing it as a complex disease. Approximately 5–7% of BC cases are attributable to germline pathogenic variants in susceptibility genes [3,4]. However, these rare variants explain only a limited portion of familial BC. The remaining inherited risk is likely distributed across other, yet-to-be-identified variants in noncoding or regulatory regions and in numerous common variants with small individual effects that, when combined, constitute a polygenic risk [5].

Polygenic risk scores (PRSs) are derived from genome-wide association studies (GWASs) and designed to identify genetic variants involved in polygenic diseases. To date, most GWASs have focused on populations of primarily European ancestry [6,7,8,9]. Assessing the performance of PRSs in more diverse populations is, therefore, crucial to guide accurate genetic risk stratification in clinical settings [10].

The Brazilian population has a unique, highly admixed genetic composition. It is mostly derived from a combination of Native Americans, Southern Europeans (Portuguese, Spanish, and Italian) who immigrated in the period 1500–1900, and Sub-Saharan Africans brought through extensive slave trading until the 1800s. More recently, from 1822 to the first half of the 1900s, other smaller waves of immigration also contributed to Brazil’s remarkable diversity, including Japanese, Lebanese, German, and Eastern Europeans [11]. Three in every four Brazilians have multiple genetic ancestries [12,13]. Given Brazil’s genetic diversity, any PRS developed in predominantly European populations requires validation before it can be used in clinical settings.

When considering genotyping approaches for both common and rare variants, several laboratory methods are available, including microarrays, whole exome sequencing (WES), and whole genome sequencing (WGS). WES offers an economical and scalable option, providing extensive coverage of coding regions while capturing both rare and common genetic variants.

In this study, we evaluated four BC PRSs [6,7,14] by performing WES in a cohort of 15,490 Brazilians. Our findings aim to clarify how effectively these PRSs perform in a population with diverse ancestry and to explore implications for future cancer risk stratification.

## 2. Materials and Methods

### 2.1. Study Population

A total of 15,490 individuals were selected for this study, including 6362 women with a history of breast cancer, and 9128 adult unphenotyped controls. Both clinical and genetic data were collected from a database of a College of American Pathologists (CAP)-accredited laboratory (Mendelics, São Paulo, SP, Brazil). All BC patients and control subjects provided informed consent for the use of retrospective anonymized data for research purposes. Samples were anonymized before analysis. Clinical records (such as BC histological type and age at diagnosis) were obtained from genetic test requisitions. The study was approved by the Institutional Review Board (IRB) (CAAE: 70112423.3.0000.0068).

### 2.2. Exome Sequencing and Imputation

Exome sequencing data were generated from buccal swabs or venous blood samples using a standard protocol for Illumina Flex Exome Prep and a custom probe set from Twist Biosciences. Sequencing was carried out on Illumina platforms, and the bioinformatics pipeline for data analysis followed the Broad Institute’s GATK best practices [15], with alignment of reads to GRCh38.

Exome imputation was based on a reference panel of 2504 individuals from the 1000 Genomes Project (1KGP) [16] on GRCh38 [17]. All regions with at least 1× coverage from exome sequencing, as well as off-target regions, were considered for imputation, which was performed using the GLIMPSE (v1.1.0) software [18].

### 2.3. Relatedness Calculation and Data Filtering

Relatedness among individuals was estimated from exome data using the somalier (v0.2.19) software [19], following the standard protocol for generating a VCF file [20]. Regarding the removal of related individuals, if two individuals had a first-degree relationship, one was randomly selected to be included in the dataset. However, if individuals had two or more first-degree relationships, all related individuals were excluded. This process resulted in a total of 211 removals. Furthermore, 73 individuals were removed from the sample due to the unavailability of files required for genome imputation, and an additional 122 individuals were removed due to low-quality imputation.

PRS analyses were performed after excluding cases and controls carrying pathogenic or likely-pathogenic (P/LP) variants in high-penetrance BC genes with OR > 5 (*BRCA1*, *BRCA2*, *TP53*, *PALB2*, and *PTEN*).

### 2.4. Polygenic Risk Score Calculation

Four BC PRSs with publicly available summary statistics from three different studies were evaluated: Khera et al. 2018 [6], with 5218 variants (PGS Catalog [21,22] ID: PGS000015); Mavaddat et al. 2019 [7] PRSs (with 313 and 3820 variants, respective PGS Catalog IDs: PGS000004 and PGS000007); and the UK Biobank (UKBB) PRS [14], obtained from variant thresholding (*p* value < 10^−5^) from summary statistics for phenotype code 20001_1002, resulting in a PRS with 7538 variants.

To address constraints associated with imputed variants, we filtered PRS variants based on their distance from the exome target regions and on their minor allele frequency (MAF). Variants with null betas (beta = 0) were removed from all PRSs. Additionally, in the original 3820-variant PRS from Mavaddat et al. [7] study, we removed a pathogenic variant of moderate penetrance in *CHEK2* (*CHEK2* p.Ile157Thr; Clinvar: RCV000144596) to avoid conflation with monogenic risk.

PRS calculation was performed using paribus (v1.0.0) in-house software at Mendelics, which computes the weighted sum of beta values, where the weights depend on the number of the individual’s alleles matching each variant of the PRS file. The sum is normalized by all positive and negative beta values so that the final score ranges from zero to one.

### 2.5. Ancestry Evaluation

Admixture [23] was used to estimate continental ancestries for all unrelated exomes with complete data. The analysis was supervised by the 1KGP samples, after removing South Asian, Oceanian, and admixed American individuals from the GRCh38 1KGP 2017 release. South Asian and Oceanian ancestries were removed because they are not a significant part of the Brazilian ancestral composition. Latin American admixed populations (Colombian, Peruvian, Puerto Rican, and Mexican) were also removed to avoid confounding with Native Americans in the same population label. The continents considered were Africa (AFR), America (AMR), East Asia (EAS), and Europe (EUR). Ancestry results were then used to group individuals according to their ancestry composition for further PRS effect-size analysis. These groups were: East Asian majority (>50% EAS, *n* = 217), Non-European majority (0–50% EUR, *n* = 760), and European majority (51–100% EUR, *n* = 13,510).

### 2.6. Genetic Principal Component Analysis (PCA)

PCA was performed by projecting exomes onto the 1KGP [16] and Human Genome Diversity Project (HGDP) [24] samples. Only variants with MAF > 1% that could have been directly genotyped using WES were included in the PCA, which was carried out in plink2 [25]. Exomes were converted to plink bfile format (bed, bim, and fam) with any duplicated variants removed. PCA projection for 10 principal components (PCs) was generated using the plink2–score method, with allele frequencies derived from the BC cohort.

### 2.7. Paired Imputed and Sequenced Genomes Analysis

Exome-imputed variants and directly sequenced variants from WGS were compared using 3119 samples from an independent Brazilian population dataset [26] that had both WES and WGS data available. The exomes were sequenced and imputed using the same methods described above. The adapted BC PRS-3820 from Mavaddat et al. [7] was calculated for both exomes (with imputation) and genomes, and their Spearman correlation was assessed using the cor.test function in R.

### 2.8. Statistical Analyses

PRS values were standardized relative to control values prior to statistical analyses. The effect size of the PRS on BC status was assessed via logistic regression, adjusting for z-scored PCs 1–10. The AUC for the full dataset was obtained in the testing split (25%) using the roc_auc function from the yardstick R package [27]. For segmentation effect sizes, individuals were classified into deciles or percentiles on left-open and right-closed intervals. To calculate the OR for a particular decile, that decile was compared against those in the median interval (40–60%), binarizing individuals as either “within the decile” (1) or “within the 40–60% interval” (0). A logistic regression was then performed on the binarized variable, correcting for PCs 1–10. A similar approach was used for percentiles to compare with Mavaddat’s [7] PRS validation. All comparisons with original studies were made using the testing set. For each ancestry proportion group, the AUC was estimated via 10-fold cross-validation using the *caret* R package [28]. ORs and CIs for *BRCA1*, *BRCA2*, *PALB2*, *TP53*, *ATM*, *CHEK2*, and the *TP53* R337H variant were obtained using the *epitools* R package [29]. All statistical tests were two-tailed, and analyses were conducted in R version 4.4.2.

## 3. Results

### 3.1. Removal of P/LP Variants Prior to PRS Assessment and Ancestry Composition of the Cohort

After removing 211 subjects with first-degree relationships, 73 with missing files necessary for imputation, and 122 with low-quality imputation, a total of 15,084 subjects remained (Supplementary Table S1). Individuals carrying pathogenic or likely-pathogenic (P/LP) variants in BC genes with ORs > 5 (*BRCA1*, *BRCA2*, *TP53*, *PALB2*, and *PTEN*) were excluded prior to PRS calculation (*n* = 629) (Methods), resulting in a final sample of 5598 women with a history of BC, and 8767 unphenotyped controls (Table 1).

**Figure 1:**
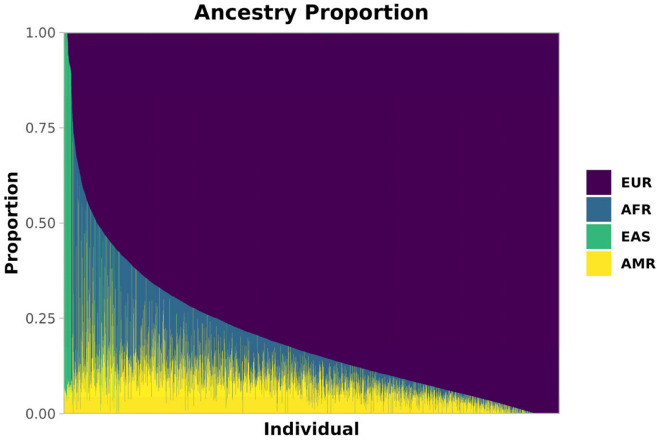
Ancestry composition of our Brazilian cohort. Estimated ancestries are shown as proportions per individual. Each thin bar represents one individual and their ancestry proportion. Europe (EUR) is purple, Africa (AFR) is blue, East Asia (EAS) is green, and America (AMR) is yellow.

The ancestry composition of this admixed cohort, obtained via Admixture [23] (Methods), is shown in Figure 1. Most individuals had a majority of European (EUR) ancestry (median 84%, SD 18%), with substantial fractions of African (AFR, median 6%, SD 12%) and Native American (AMR, median 8%, SD 7%) ancestries, along with varying EUR proportions. A smaller proportion of East Asian (EAS) ancestry was also present (median < 1%, SD 12%), predominantly contributed by 214 individuals who exceeded 70% EAS ancestry.

### 3.2. Three PRSs Identify Increased Breast Cancer Risk for Brazilian Women

Four PRS files from three studies were selected for initial effect size evaluation in this cohort: PRS_Broad_ [6], PRS_313_ [7], PRS_3820_ [7], and PRS_UKBB_ [14] (Supplementary Table S2). All PRS files were filtered to include only variants covered by the exome-imputation pipeline (Supplementary Table S5—PRS_313_, Supplementary Table S6—PRS_3820_, Supplementary Table S7—PRS_Broad_, Supplementary Table S8—PRS_UKBB_).

PRSs were then calculated for the exomes imputed into genomes and standardized for improved interpretability. The effects were corrected for the first ten PCs, and the results are reported in Supplementary Table S3.

Three of the four PRSs showed statistically significant associations with BC risk, with ORs per SD ranging from 1.35 to 1.52 (PRS_Broad_: OR 1.52, 95% CI 1.46–1.59, AUC 0.614; PRS_3820_: OR 1.43, 95% CI 1.38–1.49, AUC 0.596; PRS_313_: OR 1.35, 95% CI 1.30–1.41, AUC 0.583). The PRS_UKBB_ was not significantly associated with BC risk in this cohort (*p*-value 0.40). Model goodness of fit was highest for PRS_Broad_ (pseudo-R^2^: 0.062) and PRS_3820_ (pseudo-R^2^: 0.054).

**Figure 2:**
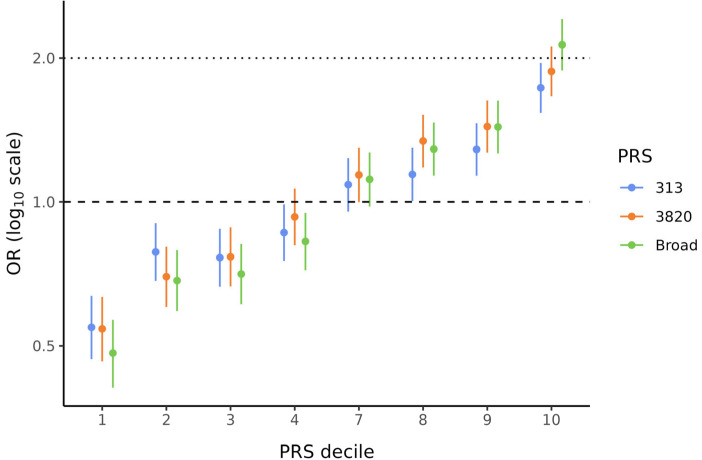
Effect sizes by decile of PRS_313_, PRS_3820_, and PRS_Broad_. Odds ratios (ORs) and 95% confidence intervals (CIs) are shown for PRS_313_ (blue), PRS_3820_ (orange), and PRS_Broad_ (green). ORs for all PRS deciles were corrected for the first ten PCs. Deciles 5 and 6 were used as references to calculate ORs for the other deciles.

Because PRS_Broad_, PRS_3820_, and PRS_313_ showed significant ORs per SD, these three scores were used to divide the data into deciles to evaluate the BC risk conferred by the PRS in each stratum. These analyses were also corrected for the first ten PCs. Figure 2 illustrates the characteristic “staircase” shape for all three PRSs. In particular, the bottom and top deciles, which are the most critical when analyzing PRS data, showed statistically significant (*p* < 0.001) and substantial effect sizes for all PRSs. ORs ranged from 0.48 to 0.55 in the lowest decile and from 1.73 to 2.13 in the highest decile (Supplementary Table S4). Notably, for PRS_Broad_, women in the top decile (90–100%) had more than twice the BC risk compared to those in the middle deciles (40–60%).

### 3.3. Effect Sizes of All PRSs Are Less Pronounced than in the Original Studies

Comparing the metrics of these PRSs in this study with those reported in their original studies offers insight into whether the scores maintain accuracy and reliability in a genetically diverse, admixed population. Overall, all PRSs showed less pronounced effects in the current cohort (OR PRS_Broad_ 1.52 vs. 1.56; PRS_3820_ 1.43 vs. 1.66, PRS_313_ 1.35 vs. 1.61) and lower classification ability (AUC PRS_Broad_ 0.61 vs. 0.69; PRS_3820_ 0.60 vs. 0.64, PRS_313_ 0.58 vs. 0.63). These differences are expected, given that the admixed Brazilian population has distinct allele frequencies and linkage disequilibrium patterns compared to the predominantly European populations used to develop and validate these PRSs. 

**Figure 3:**
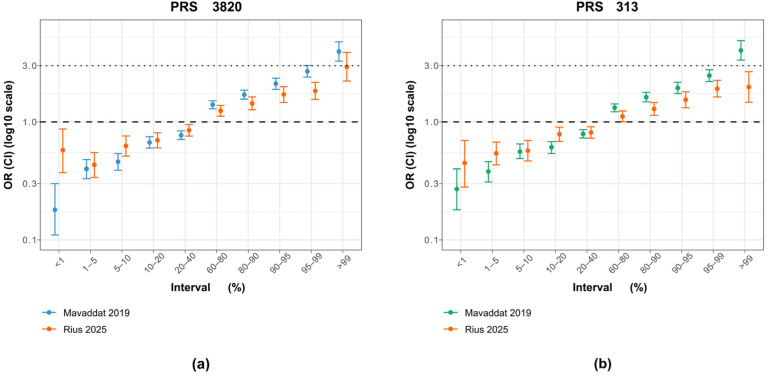
Comparison of PRS_313_ and PRS_3820_ percentile effect sizes between the original study and the Brazilian cohort. (**a**) Odds ratios (ORs) and 95% confidence intervals (CIs) for PRS_3820_ adapted in this study (orange), with 2575 variants, alongside the original PRS_3820_ from the Mavaddat et al. [7] study (blue), with 3820 variants. (**b**) Odds ratios (ORs) and 95% confidence intervals (CIs) for PRS_313_ adjusted for this study (orange), with 283 variants, alongside the original variant from the Mavaddat et al. [7] study (green), with 313 variants.

When examining the ORs by percentiles, PRS_3820_ more closely matched Mavaddat’s original findings than PRS_313_ did (Figure 3). This similarity is mainly driven by the top 1% OR, which shows a stronger effect for PRS_3820_ (OR 2.93) than for PRS_313_ (OR 1.98), bringing it closer to the original PRS_3820_ result (OR 3.95) but farther from the original PRS_313_ result (OR 4.04). Conversely, for PRS_3820_, the lowest 1% of PRS values demonstrated a smaller decrease in BC risk than in the original study, likely due to the limited sample size of that group (30 cases and 88 controls).

### 3.4. PRS_313_ and PRS_3820_ Can Stratify BC Risk in Groups with Different Ancestry Compositions

**Figure 4:**
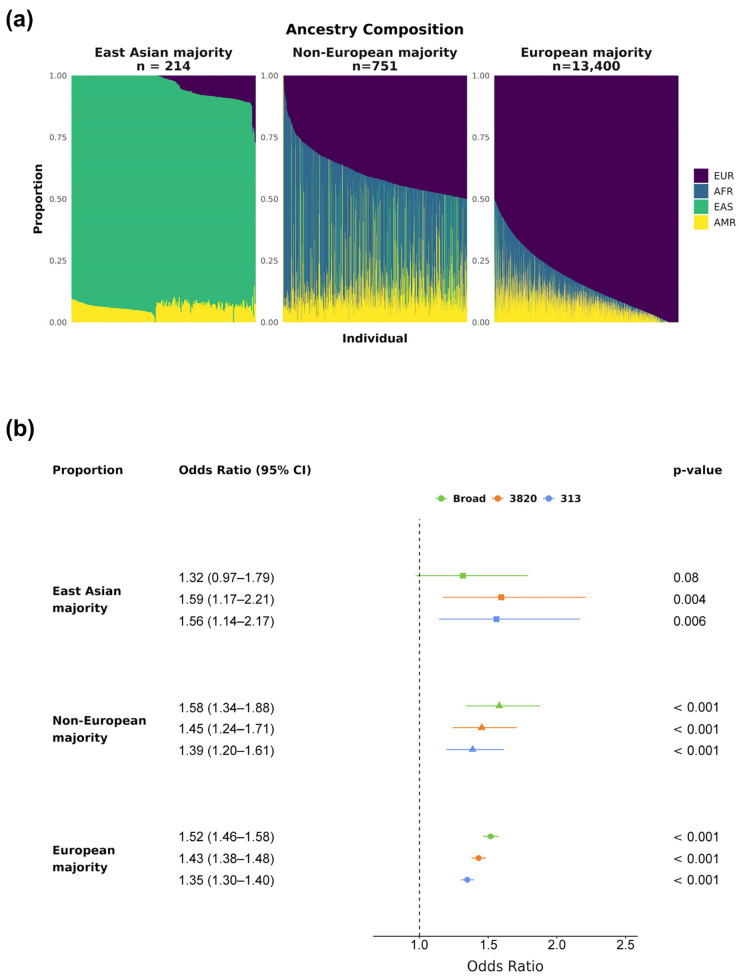
PRSs’ effect sizes by ancestry proportion. The cohort was divided into three groups based on main ancestry: East Asian, Non-European majority, and European majority. (**a**) Ancestry composition of each group, with colors representing continental ancestries for each subject: purple for EUR, blue for AFR, green for EAS, and yellow for AMR. (**b**) Breast cancer ORs and 95% CIs by PRS_Broad_, PRS_3820_, and PRS_313_ SD for the three ancestry groups (East Asian majority in squares, Non-European majority in triangles, and European majority in circles). All *p*-values displayed were corrected for multiple hypothesis testing via the Bonferroni method.

Because the majority of individuals in this cohort exhibit mostly European ancestry, we evaluated the PRS effect sizes across groups with different ancestry proportions (Figure 4a, Methods). All three bins showed statistically significant (*p*-value < 0.001) ORs above 1.35 per SD for PRS_313_ and PRS_3820_, indicating a positive association between PRS value and elevated BC risk across all ancestry groups (Figure 4b). PRS_Broad_ was also significant for both European-related groups (*p*-value < 0.001), providing the highest effect sizes among the evaluated PRSs (Non-European majority: OR 1.58, 95% CI 1.34–1.88; European majority: OR 1.52, 95% CI 1.47–1.58). However, PRS_Broad_ did not reach statistical significance for the East Asian majority group (*p*-value 0.08), suggesting it may be less suitable for use in a Brazilian admixed population. 

### 3.5. Imputation Is a Reliable Tool for PRS Assessment

Subsequent analyses focused on PRS_3820_. A correlation of 0.74 (*p*-value < 2.2 × 10^−16^) was found when comparing PRS_3820_ values from exomes (imputed) versus those from whole-genome sequencing in 3119 individuals from an independent Brazilian dataset (Supplementary Figure S1a). Decile classification also showed strong agreement, with 57% of individuals in the top and bottom deciles overlapping in both datasets (Supplementary Figure S1b). Notably, most individuals assigned to different deciles were found in adjacent deciles, reflecting consistent results from both imputed and directly sequenced data.

### 3.6. PRS_3820_ Top Decile OR Is Comparable to That of a Moderate Risk BC Gene

**Figure 5:**
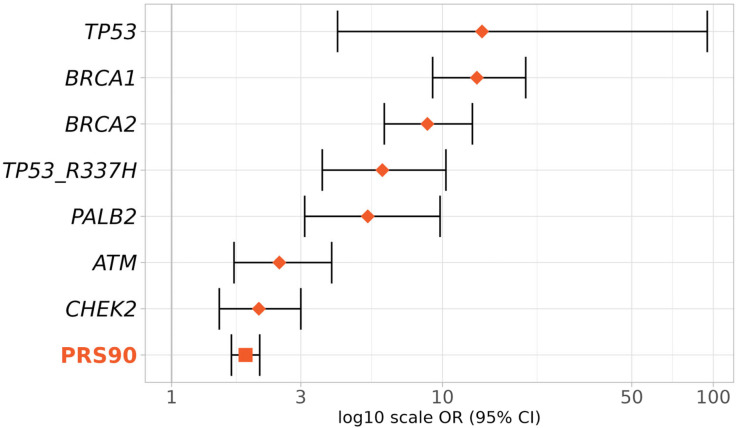
Effect sizes of the top deciles of PRS and BC genes in BC risk. Effect sizes (ORs and 95% CIs) were obtained according to the presence of pathogenic variants in the genes *TP53*, *BRCA1*, *BRCA2*, *PALB2*, *ATM*, and *CHEK2*, or inclusion in the 90th to 100th percentiles of PRS_3820_ (PRS90). Diamonds represent ORs given by BC genes (monogenic) and the square represents the OR given by the PRS (polygenic).

To evaluate the PRS_3820_ effect size relative to known high- and moderate-risk BC genes, we compared the OR for the top PRS_3820_ decile (PRS90) with that of pathogenic variants in *TP53*, *BRCA1*, *BRCA2*, *PALB2*, *ATM*, and *CHEK2* genes plus the pathogenic variant R337H of the *TP53* gene (Figure 5). 

As expected, the highest BC risks were conferred by *TP53*, *BRCA1*, and *BRCA2* (*TP53* OR 14, 95% CI 4.1–95; *BRCA1* OR 13.4, 95% CI 9.2–20.3; *BRCA2* OR 8.8, 95% CI 6.1–12.9, respectively). The OR for the top PRS_3820_ decile (1.9, 95% CI 1.7–2.1) was slightly lower than that of the moderate-risk BC genes *ATM* (OR 2.5, 95% CI 1.7–3.9) and *CHEK2* (OR 2.1, 95% CI: 1.5–3), but still in a similar range.

## 4. Discussion

In the present study, we validated two breast cancer PRSs originally developed from Europeans in a highly admixed Brazilian population. The PRSs adapted from Mavaddat et al. [7], consisting of 283 (PRS_313_) and 2575 (PRS_3820_) variants, showed statistically significant risk prediction both per PRS standard deviation (SD) and in the top decile compared with the middle deciles (*p*-values < 0.001). PRS_3820_ demonstrated the best performance, with an odds ratio (OR) of 1.43 per SD (95% CI 1.38–1.49) and 1.88 for the top decile (95% CI 1.66–2.12). Furthermore, this PRS yielded an OR per SD of 1.43 or higher across different ancestry compositions (East Asian majority: OR 1.59, 95% CI 1.17–2.21, *p* value 0.004; Non-European majority: OR 1.45, 95% CI 1.24–1.71, *p*-value < 0.001; and European majority: OR 1.43, 95% CI 1.38–1.48, *p*-value < 0.001), highlighting its suitability for Brazil’s diverse population. 

The best-performing PRS in this study is based on the work of Mavaddat et al. (2019) [7], who developed and validated a 3820-variant score to assess invasive BC risk. For all BC subtypes (ER+ and ER−), they reported an OR of 1.71 per SD (95% CI: 1.64–1.79) in the validation set (*n* = 29,751; cases = 11,428) and an OR of 1.66 per SD (95% CI 1.61–1.70) in the prospective set (*n* = 190,040; cases = 3215). These values exceed those of the widely used 313-variant PRS (OR 1.65 per SD; CI 1.59–1.72 in the validation set). However, they included a pathogenic variant in the *CHEK2* gene in their PRS and focused solely on invasive BC, which may have led to higher OR values.

A study by Liu and colleagues [30] evaluated another modification of the same 3820-variant PRS (originally from Mavaddat et al. [7]) in African, Latin, and European populations. Their findings showed an effect size of this PRS of 1.40 per SD in a European sample (*n* = 33,594), a figure very similar to our result in a Brazilian sample (OR 1.43 per SD; *n* = 14,365). Liu et al. [30] specifically included women with in situ ductal BC in addition to those with invasive BC, which they attributed to the lower OR compared with the original study. Our study does not distinguish between invasive and in situ BC; therefore, we hypothesize that both types are included, which, together with differences in genetic population structure, may explain the lower OR value relative to the original study.

Subtype-specific analyses of breast cancer, particularly for estrogen receptor–positive (ER+) and estrogen receptor–negative (ER−) cases, have been conducted by Mavaddat et al. (2019) [7] and others [31,32,33]. Due to limited subtype information in our cohort, we were unable to perform a similar analysis. Nevertheless, previous studies have demonstrated that breast cancer PRSs exhibit meaningful classification performance and effect sizes across subtypes [7,34]. These findings support the relevance and utility of our PRS results, even in the absence of subtype-specific stratification. Future research should aim to address this limitation to improve subtype-specific understanding.

Gene-environment interactions have been investigated in several complex diseases, including BC, to elucidate how a polygenic risk score (PRS) may modulate or be modulated by environmental exposures [35,36,37,38,39,40]. This interplay is particularly complex in admixed populations, whose unique genetic architecture and phenotypic variability may influence the magnitude and direction of gene-environment effects. In BC, one relevant question is whether there might be an environmental factor that has a multiplicative influence on an individual’s PRS. Although the present study did not explicitly evaluate environmental factors, other investigations have explored how PRS interacts with key lifestyle factors in BC risk. Studies using the UK Biobank (UKBB) and Breast Cancer Association Consortium (BCAC) cohorts have found that PRS generally acts additively rather than multiplicatively with lifestyle factors such as weight, alcohol intake, physical activity, oral contraceptive use, and hormone replacement therapy [37,38,39,40]. For example, Arthur et al., 2020 reported that among individuals with a high PRS, those who also maintained a healthier lifestyle had a substantially lower BC risk (hazard ratio: 0.73) than those in a less healthy lifestyle category [37]. Furthermore, an investigation assessing alcohol consumption and a BC PRS in the UKBB observed no significant multiplicative interaction in either White or Black individuals, implying that, at least for alcohol consumption, ethnically diverse populations did not exhibit a strong gene-environment effect [40]. While additional research, particularly involving admixed populations, is certainly needed, current large-scale studies suggest that any potential lifestyle interactions with PRS do not modify overall risk assessment.

All of our PRS values were calculated using a novel methodology involving exome imputation. This approach has proven highly effective for PRS calculation and BC risk assessment in our study and could be particularly appealing to laboratories already performing exome sequencing as a cost-effective method to identify P/LP variants for BC. Multiple studies have compared low-pass genome sequencing and array-based methods for various applications, including pharmacogenetics, GWASs, and PRS calculations [41,42]. Li et al. [42] reported improved accuracy for polygenic risk prediction of imputed low-pass genomes compared with array imputation for both coronary artery disease and BC. Despite the slight difference we observed between PRS values calculated from sequenced genomes and those from imputed exomes (ρ = 0.74), decile classification showed satisfactory concordance for the extreme deciles (1st and 10th), which are most important for determining decreased or increased risk. Unfortunately, it was not possible to evaluate the predictive power of PRS values derived from the genomes of BC patients because paired exome and genome data were unavailable.

In addition, another group has published a PRS analysis in a smaller independent cohort of Brazilian BC patients [43]. They reported a classification ability in two Brazilian cohorts from the PRS 313 (AUC: 0.66 and 0.62) that is similar to the one assessed in a UKBB cohort (0.63). Another study has evaluated the classification ability of all BC PRSs available in the literature (*n* = 120) in 931 cases and 1048 controls of an independent Brazilian cohort [44]. They have identified 109 PRSs statistically associated with ER+/HER2- BC risk, where the top five have good classification abilities (AUC 0.64–0.65) and important effect sizes (OR per SD 1.74–2.09).

Among familial BC patients, approximately 25% have a P/LP germline variant [45]. In the Brazilian population, a robust study with 1663 BC patients evaluated for hereditary BC using multigene panel testing reported that 20.1% had P/LP germline variants [46,47]. Additionally, a 2017 study reported that 18% of hereditary BC can be explained by a polygenic effect of variants discovered in GWAS [48]. Taken together, these findings highlight the contribution of both germline variants and polygenic factors to hereditary BC. In this study, we demonstrate that polygenic risk scores (PRSs), developed in European cohorts, can reliably be used to identify BC risk in Brazilian women.

By December 2024, three commercially available tests with BC PRSs in Brazil had been launched [49,50,51]. None offer clinical recommendations. Future clinical studies are, therefore, needed to determine the most effective strategies for integrating PRSs into BC screening in the Brazilian population.

## 5. Conclusions

In conclusion, this study validated PRS_3820_ and PRS_313_ in the Brazilian population, demonstrating that PRS_3820_, in particular, can reach effect sizes comparable to certain moderate-risk monogenic variants. While these results highlight the potential value of PRS in enhancing risk stratification for BC, further studies are necessary to determine how such data should be integrated into clinical practice and to guide the development of Brazil-specific recommendations for managing individuals with higher polygenic risk.

## Figures and Tables

**Table 1:** Demographics of the patients and controls in the BC dataset used for PRS evaluation.

		Case	Control	Total	*p*-Value
	Total	5598	8767	14,365	-
Sex	Female	5598	4187	9785	-
	Male	-	4580	4580	-
Age, mean (SD)	Total	49.8 (11.6)	41.6 (13.3)	44.8 (13.3)	0.000
	Female	49.8 (11.6)	41.9 (13.7)	46.4 (13.1)	0.000
	Male	-	41.3 (12.9)	41.3 (12.9)	-

*p*-values were obtained from two-tailed *t* tests.

## Data Availability

The original contributions presented in this study are included in the article/Supplementary Material. Individual case and control data are not publicly available due to the confidentiality of the consent agreement signed by all those included in the study.

## Supplementary Materials

The following supporting information can be downloaded at https://www.mdpi.com/article/10.3390/diagnostics15091098/s1: Figure S1: Correlation of PRS_3820_ values for exomes with imputation and genomes; Table S1: Demographics of cases and controls and BC characteristics; Table S2: Number of individuals and variants of the four PRSs evaluated in this study; Table S3: Metrics for all PRSs evaluated; Table S4: PRS_Broad_, PRS_3820_ and PRS_313_ decile odds ratios and confidence intervals; Table S5: Variants of PRS_313_; Table S6: Variants of PRS_3820_; Table S7: Variants of PRS_Broad_; Table S8: Variants of PRS_UKBB_.

## Abbreviations

The following abbreviations are used in this manuscript:

PRS	Polygenic Risk Score
BC	Breast Cancer
OR	Odds Ratio
GWAS	Genome Wide Association Studies
WES	Whole Exome Sequencing
WGS	Whole Genome Sequencing
CAP	College of American Pathology
IRB	Institutional Review Board
CAAE	Certificado de Apresentação de Apreciação Ética
GATK	Genome Analysis Toolkit
1KGP	1000 Genomes Project
VCF	Variant Call Format
P/LP	Pathogenic or Likely-Pathogenic
PGS	Polygenic Score
ID	Identification
UK	United Kingdom
MAF	Minor Allele Frequency
AFR	Africa
AMR	America
EAS	East Asia
EUR	Europe
HGDP	Human Genome Diversity Project
PCA	Principal Components Analysis
PC	Principal Component
AUC	Area Under The Curve
CI	Confidence Interval
SD	Standard Deviation
UKBB	UK Biobank
PRS90	90th to 100th Percentile of Polygenic Risk Score
ER	Estrogen Receptor
CNPq	Conselho Nacional de Desenvolvimento Científico e Tecnológico
