# Supplementary material for: A Breast Cancer Polygenic Risk Score Validation in 15,490 Brazilians Using Exome Sequencing
Source: Diagnostics (Basel). 2025 Apr 25;15(9):1098. doi: 10.3390/diagnostics15091098 (PMC12071591; doi:10.3390/diagnostics15091098)
Supplement: Supplementary file 1 [file diagnostics-15-01098-s001.zip › Supplementary Information - Tables and Figure.pdf]

Supplementary Information

Supplementary Tables

Table S1. Demographics of cases and controls and BC characteristics.

		Case	Control	Total	p.value
	Total	6206	8878	15084	-
**Sex**	F	6206	4241	10447	-
	M	-	4637	4637	-
**Age**	Total	49.5 (11.7)	41.6 (13.3)	44.9 (13.2)	0.000
	F	49.5 (11.7)	42 (13.7)	46.5 (13.1)	0.000
	M	-	41.3 (12.9)	41.3 (12.9)	-
**P/LP variants***	No	5598	8767	14365	-
	Yes	608	111	719	-
**Subtype****	HER2+	162	-	162	-
	HR+	306	-	306	-
	Triple Negative	1025	-	1025	-
	No information	4713	-	4713	-
**Multiple Breast Tumors**	No	5729	-	5729	-
	Yes	477	-	477	-

* Pathogenic variants on the genes *BRCA1*, *BRCA2*, *TP53*, *PALB2*, and *PTEN* were evaluated to classify individuals in this category.

** For multiple tumors with information we have selected the subtype of the most recent tumor.

Table S2. Number of individuals and variants of the four PRSs evaluated in this study.

Study	PRS name	PGS Catalog ID	Total in discovery sample	Cases	Controls	Original variants
Khera et. al, 2018	Broad	PGS000015	228951	122977	105974	5,218
Mavaddat et. al, 2019	313	PGS000004	169092	94075	75017	313
Mavaddat et. al, 2019	3820	PGS000007	169092	94075	75017	3820
UKBB (phenotype 20001_1002)	UKBB	-	194153	7968	186185	7538

* Number of variants after adjustment for imputation coverage.

Table S3. Metrics for All PRSs Evaluated.

PRS	OR	p value	Lower 2.5% CI	Upper 97.5% CI	AUC	Nagelkerke pseudo-R^2
Broad	1.52	6.10E-82	1.46	1.59	0.614	0.062
3820	1.43	1.02E-68	1.38	1.49	0.596	0.054
313	1.35	1.64E-49	1.3	1.41	0.583	0.042
UKBB	1.02	0.40	0.98	1.06	0.545	0.014

* AUC: Area under the receiving operated curve, evaluating the performance of PRS only, without covariates, on BC classification.
** Variance explained by PRS: calculated as a difference of Nagelkerke's pseudo-R^2 value obtained in a model with and without the evaluated PRS.

Table S4. PRS_{Broad}, PRS_{3820} and PRS_{313} decile Odds Ratios and Confidence Intervals.

**Broad**				
**ORs**	**lower 95% CI**	**upper 95% CI**	**p value**	**decile**
0.48	0.41	0.57	1.90E-18	10%
0.69	0.59	0.79	4.39E-07	20%
0.71	0.61	0.82	2.64E-06	30%
0.83	0.72	0.95	0.0072	40%
1.12	0.98	1.27	0.1018	70%
1.29	1.14	1.47	0.0001	80%
1.44	1.26	1.63	2.46E-08	90%
2.13	1.88	2.41	3.97E-33	100%

**3820**				
**ORs**	**lower 95% CI**	**upper 95% CI**	**p value**	**decile**
0.54	0.46	0.63	1.37E-14	10%
0.70	0.60	0.81	1.16E-06	20%
0.77	0.67	0.89	2.66E-04	30%
0.93	0.81	1.07	0.2990	40%
1.14	1.00	1.30	0.0514	70%
1.34	1.18	1.52	0.0000	80%
1.44	1.27	1.63	1.44E-08	90%
1.88	1.66	2.12	1.19E-24	100%

313				
**ORs**	**lower 95% CI**	**upper 95% CI**	**p value**	**decile**
0.55	0.47	0.64	7.93E-15	10%
0.79	0.68	0.90	6.81E-04	20%
0.77	0.67	0.88	1.66E-04	30%
0.86	0.75	0.99	0.0330	40%
1.09	0.96	1.24	0.2072	70%
1.14	1.00	1.30	0.0429	80%
1.29	1.14	1.46	8.62E-05	90%
1.73	1.54	1.95	3.56E-19	100%

Supplementary Figures

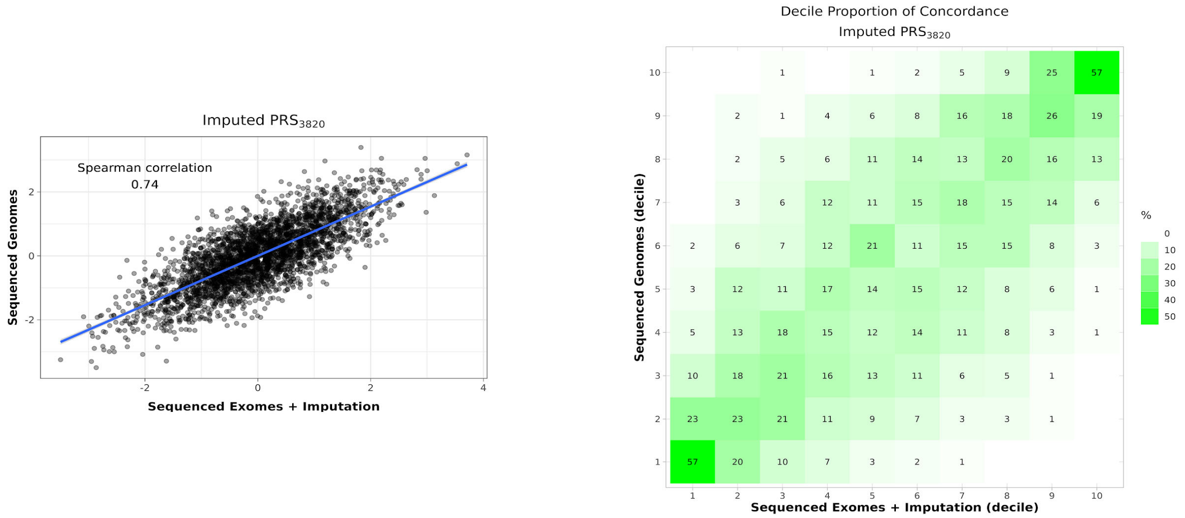

(a) **(b)**

Figure S1. Correlation of PRS_{3820} values for exomes with imputation and genomes. (**a**) Correlation between PRS values for exomes with imputation and sequenced genomes. Spearman correlation rho value is shown in the plot area. (**b**) Heatmap showing the percentage of samples classified in a determined combination of deciles between exomes with imputation (x axis) and genomes (y axis). Proportion expressed in percentage per decile.
