# Supplementary figures and images for: A Breast Cancer Polygenic Risk Score Validation in 15,490 Brazilians Using Exome Sequencing
Source: Diagnostics (Basel). 2025 Apr 25;15(9):1098. doi: 10.3390/diagnostics15091098 (PMC12071591; doi:10.3390/diagnostics15091098)

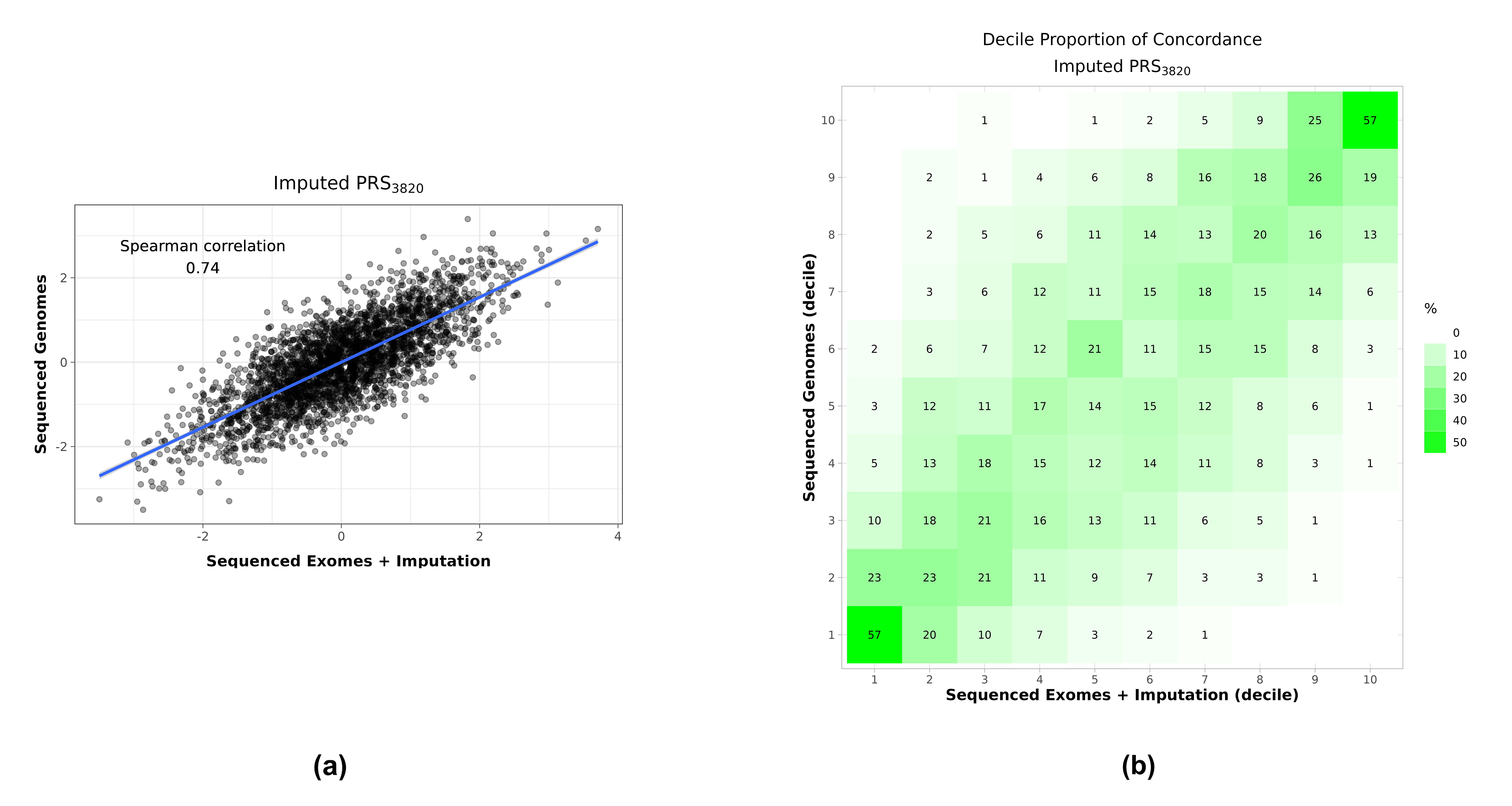

Supplement: Supplementary file 1 [file diagnostics-15-01098-s001.zip › Figure S1 Correlation of PRS3820 values for exomes with imputation and genomes.png]
